# Highly sensitive genotyping of MTHFR C677T polymorphisms using a novel RPA-LDR-qPCR assay: RPA-LDR-qPCR for MTHFR C677T genotyping

**DOI:** 10.3724/abbs.2022151

**Published:** 2022-10-20

**Authors:** Xinxin Si, Qinghua Gu, Chenjie Zhao, Xiao Zhang, Tingting Xiao, Yu Li, Wei Ying, Song Gao

**Affiliations:** 1 Jiangsu Key Laboratory of Marine Biological Resources and Environment Jiangsu Key Laboratory of Marine Pharmaceutical Compound Screening Co-Innovation Center of Jiangsu Marine Bio-industry Technology School of Pharmacy Jiangsu Ocean University Lianyungang 222005 China; 2 Department of Pathology Jiangsu Province Hospital of Chinese Medicine Nanjing 210029 China

The 5,10-methylenetetrahydrofolate reductase (MTHFR) is an important enzyme involved in the metabolism of folic acid, synthesis of DNA, and DNA methylation. The genetic single nucleotide polymorphisms (SNPs) of MTHFR caused by the C677T mutation have become an important clinical indicator related to folic acid intake in relevant populations [1]. The C677T mutation, replacing cytosine (C) by thymine (T) at the site, causes the change of encoded amino acid from alanine to valine, reducing the enzyme activity and heat resistance capacity. In the MTHFR polymorphism of the hybrid type (CT), the enzyme activity is reduced by 30%, while in the polymorphism of the homozygous type (TT), the activity is reduced by more than 65%, causing various degrees of folic acid metabolism disorders [2]. Therefore, MTHFR polymorphisms are important information for clinicians to make personalized nutrition supplementation or treatment decisions.

**Figure 1:**
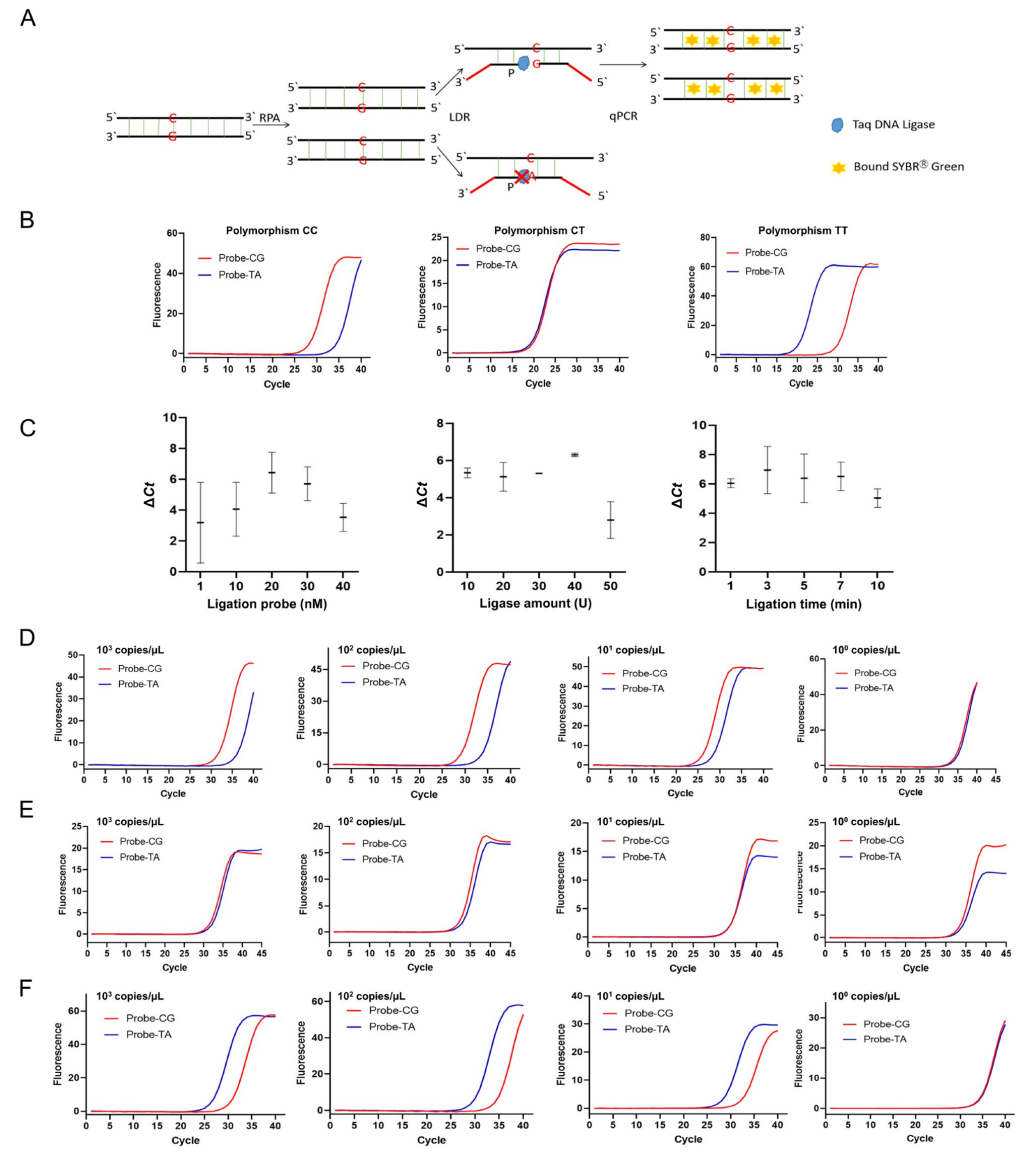
Figure 1 Establishment of the RPA-LDR-qPCR assay for genotyping MTHFR polymorphisms (A) Schematic diagram of the RPA-LDR-qPCR assay. DNA backbones are indicated as horizontal lines with 5′ and 3′ ends marked. Base pairs are indicated as vertical lines. The bases at site 677 are indicated. Partial sequences of the ligation probes in the LDR step are artificially designed and shown in red. This diagram shows the RPA-LDR-qPCR process of the wild-type allele only. (B) Result of the proof-of-principle experiment. Fluorescence curves of the qPCR step of the assay testing the 3 types of polymorphisms are shown. For the LDR step, 20 nM ligation probe, 40 U ligase, and 3 min ligation time were used. The curves represent results from three independent experiments. (C) Optimization of LDR conditions, including the ligation probe concentration, ligase amount, and ligation time. The optimization aimed at a larger difference in the Ct values (Δ Ct) between the 2 qPCR curves from the LDR of the 2 probe sets. The error bars represent the results from three independent experiments. (D‒F) The sensitivity of the RPA-LDR-qPCR assay. Fluorescence curves of the qPCR step of the assay testing CC (D), CT (E), and TT (F) polymorphisms with sample concentrations at 10 3, 10 2, 10 1, and 10 0 copies/μL are shown. For the LDR step, the optimized condition (20 nM ligation probe, 40 U ligase, and 3 min ligation time) was used. The curves represent results from three independent experiments.

For nucleic acid analysis and genotyping, sequencing is always the gold standard method but not necessarily the best choice because of its high cost and long turnover time. The most widely used method in clinical practice for genotyping MTHFR C677T polymorphisms is fluorescence probe PCR [3]. However, several flaws of the fluorescence probe PCR method reduce its accuracy. For example, it has a detection limit of 200 copies/μL, which could leave some low-content samples undetectable, and it requires high quality samples, which could lead to ambiguous results for samples not processed well. To establish a MTHFR polymorphism genotyping assay with higher sensitivity and lower sample quality requirement, we combined recombinase polymerase amplification (RPA) [4] and ligase detection reaction (LDR) [5] with qPCR technology ( Figure 1A).

The DNA fragment containing the 677 site is exponentially amplified with RPA, and 2 sets of ligation probes are used in the subsequent LDR. The bases at the 3′ ends of the 2 upstream probes are complementary to the wild-type base (C) and the mutant base (T) at site 677. The 5′ end of the downstream probe is labeled with a phosphate group to facilitate ligation. When the wild-type fragment is amplified, it serves as the ligation template for one of the ligation probe sets, while the other probe set cannot be ligated, and *vice versa*. The ligated probe set is analyzed with fluorescence qPCR, and the fluorescent signal from a certain probe set indicates a wild-type or mutant base at the 677 site of the original fragment. With a series of optimization and validation, the RPA-LDR-qPCR assay established in this study showed high sensitivity and good performance.

A 117-bp DNA fragment of the *MTHFR* gene (GenBank accession No. NM_005957.5) containing the MTHFR C677T polymorphism site (C/T, rs1801133) was selected for the initial RPA amplification. The sequences of the forward and reverse primers used for RPA were 5′-CTGACCTGAAGCACTTGAAGGAGAAGGT-3′ and 5′-TGCCCATGTCGGTGCATGCCTTCAC-3′, respectively. For the LDR step, the 2 upstream ligation probes in the 2 probe sets had one base difference at the 3′ end. The sequence of the upstream probe CG was 5′-CGGTTTTGGCGCAGTGACGCTGCGTGATGATGAAATCGG-3′, which complemented the wild-type base (C) at site 677. The sequence of the upstream probe-TA was 5′-CGGTTTTGGCGCAGTGACGCTGCGTGATGATGAAATCGA-3′, which complemented the mutant base (T) at site 677. The sequence of the downstream probe was the same in the 2 probe sets, 5′-PHO-CTCCCGCAGACACCTTCTCCAGATAGCCAACCCGAGCGCCT-3′, with a phosphate group at the 5′ end to facilitate ligation. Partial sequences of the upstream and downstream probes were artificially designed and used for the subsequent fluorescence qPCR analysis, which are shown in italics above. The sequences of the forward and reverse primers for fluorescence qPCR were 5′-CGGTTTTGGCGCAGTGACG-3′ and 5′- AGGCGCTCGGGTTGGCTATCT-3′, respectively. The primers and probes were synthesized by General Biology (Hefei, China).

Plasmids containing the MTHFR C677T polymorphism fragments were constructed and used as DNA standards in this study. DNA fragments of the *MTHFR* gene containing the MTHFR polymorphisms (353 bp) were PCR-amplified with forward and reverse primers as follows: 5′-CGGTTTTGGCGCAGTGACG-3′ and 5′-CTGGGAAGAACTCAGCGAACT-3′. The amplification products were inserted into the pMD18-T vector and propagated in DH5α competent cells. The plasmids were extracted from single colonies and confirmed by DNA sequencing (General Biology). The plasmids containing wild-type or mutant MTHFR polymorphism fragments were used as wild-type or mutant DNA standards. Quantification was performed with a Qubit 4 fluorometer (Thermo Fisher Scientific, Wilmington, USA), and the copy numbers were calculated based on the plasmid size (3045 bp).

The RPA reaction was performed as described in the manufacturer’s instructions using the RAA-Basic Nucleic Acid Amplification Reagent (Hangzhou ZC Bio-Sci & Tech Co. Ltd, Hangzhou, China). Using 1 μL of the DNA sample as the template, we performed the amplification at 37°C for 30 min. One microliter of the RPA product was applied to the subsequent LDR. The LDR mixture of 10 μL contained 1 μL of each of the upstream and downstream ligation probes (1‒40 nM), 10‒50 U of *Taq* DNA ligase (Sangon Biotech, Shanghai, China), and 1 μL of 10× *Taq* DNA ligase buffer. The mixture was denatured at 95°C for 3 min and incubated at 60°C for 1‒10 min for ligation. One microliter of the LDR product was analyzed by fluorescence qPCR. The 20-μL qPCR mixture contained 10 μL of the MonAmp SYBR Green I qPCR Mix (Monad Biotech, Wuhan, China) and 0.4 μL of each qPCR primer (10 μM) and was analyzed on a LightCycler 480 qPCR machine (Roche, Basel, Switzerland) with 40‒45 cycles of 95°C for 10 s and 60°C for 30 s.

The 3 types of MTHFR C677T polymorphisms are the homozygous type “CC”, the heterozygous type “CT”, and the homozygous type “TT” [6]. In the proof-of-principle experiment, 10 ^4^ copies of the wild-type DNA standard, the mutant DNA standard, and the 1:1 molar ratio mix of the two were tested representing CC, TT and CT polymorphisms, respectively. Each polymorphism was tested with the 2 probe sets ( Figure 1B). For polymorphism CC, the fluorescence qPCR curve from the LDR of the Probe-CG set had a distinctly lower *Ct* value than the curve from the LDR of the Probe-TA set. The same result was obtained for polymorphism TT. For polymorphism CT, the 2 qPCR curves from the LDRs of the Probe-CG and Probe-TA sets had similar *Ct* values. These results suggested that the qPCR curves of the RPA-LDR-qPCR assay could indicate MTHFR polymorphism genotyping.

LDR was the key step to distinguish the wild-type or mutant base at site 677. The reaction condition was optimized with the ligation probe concentration, the ligase amount, and the ligation time, referring to the largest difference in the *Ct* values (Δ *Ct*) between the 2 qPCR curves from the LDR of the 2 probe sets ( Figure 1C). The wild-type DNA standard (10 ^4^ copies) representing the CC polymorphism was used for the optimization. The best reaction conditions were determined to be 20 nM ligation probe, 40 U ligase, and 3 min ligation time at 60°C.

The sensitivity of the RPA-LDR-qPCR assay was evaluated for the 3 types of polymorphisms. The wild-type DNA standard, the mutant DNA standard, and the 1:1 molar ratio mix of the two, representing CC, TT and CT polymorphisms, at concentrations 10 ^3^, 10 ^2^, 10 ^1^, and 10 ^0^ copies/μL were tested in the RPA-LDR-qPCR assay ( Figure 1D‒F). The results showed that distinct genotyping could be observed for sample concentrations as low as 10 copies/μL for all 3 polymorphisms. The fluorescence signals were distinguishable from the no-template controls, as no signal curve was observed within 45 qPCR cycles when no DNA standard was input to the assay.

The RPA-LDR-qPCR assay was validated with 40 clinical samples provided by Jiangsu Province Hospital of Chinese Medicine (Nanjing, China). The templates were prepared by Jiangsu Province Hospital of Chinese Medicine using the internal blood sample processing protocol. The reference fluorescence probe PCR method followed the instructions of the Human MTHFR Gene Polymorphism Detection kit (YZY Medical Technology, Wuhan, China) that had been extensively used for MTHFR C677T polymorphism genotyping in clinical practice. The results indicated that 38 out of the 40 samples gave consistent genotyping results between the RPA-LDR-qPCR assay and the reference fluorescence probe PCR method, while 2 samples gave undetectable results with reference fluorescence probe PCR but were determined for polymorphism by RPA-LDR-qPCR ( Table 1). The polymorphism types of the 2 samples were further confirmed by DNA sequencing (General Biology).

**Table:** **Table 1** Analysis of MTHFR C677T polymorphisms in clinical samples

Sample No.	Genotyping result
	Fluorescence probe PCR	RPA-LDR-qPCR
1	CC	CC
2	CC	CC
3	TT	TT
4	CC	CC
5	TT	TT
6	CT	CT
7	CT	CT
8	CC	CC
9	CT	CT
10	TT	TT
11	CC	CC
12	TT	TT
13	TT	TT
14	CT	CT
15	CC	CC
16	CT	CT
17	CC	CC
18	CT	CT
19	CT	CT
20	CC	CC
21	CC	CC
22	TT	TT
23	CT	CT
24	Undetectable ^1^	CC
25	TT	TT
26	CT	CT
27	CT	CT
28	CT	CT
29	CT	CT
30	TT	TT
31	Undetectable ^1^	CC
32	CC	CC
33	CT	CT
34	CC	CC
35	TT	TT
36	TT	TT
37	CT	CT
38	CC	CC
39	CT	CT
40	CC	CC

^1^Polymorphism type was further confirmed by DNA sequencing.

The sensitivity of the RPA-LDR-qPCR assay established in this study for genotyping of MTHFR C677T polymorphisms achieved 10 copies/μL, which is ~20-fold higher than the extensively used fluorescence probe PCR [ 3, 7] , without significantly complicating the assay procedure. Improving sensitivity and tolerance for raw samples has become a major goal in the development of novel SNP genotyping assays [8]. The value of the RPA-LDR-qPCR assay in this aspect was demonstrated by testing clinical samples. The RPA step provides another round of amplification of the target, and LDR has been used to detect low copies of DNA mutations [9]. The combination of RPA and LDR with qPCR indeed significantly improved the assay sensitivity. The isothermal amplification feature of RPA does not require sophisticated thermal cyclers. Moreover, RPA can tolerate amplification inhibitors and deal with crude samples [10], which lowers the sample quality requirement of the assay. As the ability to process blood samples varies in medical facilities worldwide, an assay with improved sensitivity and sample tolerance has its value. In the RPA-LDR-qPCR assay, although two additional steps are added compared to the fluorescence probe PCR method, it retains acceptable convenience while providing much higher sensitivity, which is important for accurate diagnosis in clinical practice.

In conclusion, we established a highly sensitive RPA-LDR-qPCR assay for genotyping MTHFR C677T polymorphisms. It provides an alternative tool for a more accurate and reliable diagnosis of folic acid metabolism disorders and supports relevant personalized medicine.
